# The current status and emerging trends in the application of precision nutrition for the comprehensive, lifecycle-based management of chronic liver diseases

**DOI:** 10.3389/fnut.2026.1827158

**Published:** 2026-06-12

**Authors:** Mingli Lou, Xinrui Zhang, Sa Xiao, Mingxia Zhou, Hailing Qiao, Feng Wang, Yurong Xing

**Affiliations:** 1 International Medical Department, The First Affiliated Hospital of Zhengzhou University, Zhengzhou, China; 2 Henan University, Zhengzhou, China; 3 International Medical Department, Institute of Clinical Pharmacology, Zhengzhou University, Zhengzhou, China

**Keywords:** chronic liver disease, health management, hepatitis, hepatocellular carcinoma, liver cirrhosis, metabolic dysfunction–associated fatty liver disease, precision nutrition

## Abstract

Chronic liver disease (CLD) is defined as a progressive deterioration of hepatic function lasting for more than 6 months, which poses a significant challenge to global public health due to its high rates of morbidity, disability, and mortality. Traditional nutritional management often fails to meet the individualized needs of patients at different stages of disease progression and throughout their lifespan. In this context, precision nutritional intervention—a dietary approach—has garnered increasing attention in the management of CLD, due to its core advantages of dynamic optimization management strategies based on individual metabolic characteristics. This study provides a narrative review and critical analysis of the theoretical foundations, current applications, and recent advancements in precision nutrition interventions for managing CLD. Additionally, it highlights existing gaps, practical challenges, and potential directions for future research and clinical applications. The main objective is to demonstrate how multidisciplinary collaborative innovation covering hepatology, nutrition, bioinformatics, and health technology, and the optimization of supporting technologies, can accelerate the clinical transformation and widespread application of precision nutrition interventions in CLD. Ultimately, this approach aims to provide robust evidence-based decision support for developing an efficient, highly individualized health management system that encompasses prevention, treatment, and rehabilitation, thereby providing substantial clinical and public health benefits.

## 1 Introduction

Chronic liver disease (CLD) is classified as a significant form of chronic non-communicable disease, encompassing conditions such as viral hepatitis, metabolic-associated fatty liver disease (MAFLD/NAFLD), alcoholic liver disease (ALD), and cirrhosis. These diseases have high global incidence and mortality rates, making them a serious public health challenge (1). Epidemiological data estimate that approximately 1.8 billion individuals worldwide are affected by CLD. Moreover, the disease burden has shown a persistent upward trend, severely compromising patient health and quality of life (QoL) while also placing substantial pressure on healthcare and social security systems (2). Nutritional and metabolic disorders are key pathophysiological features in the onset and progression of CLD. These disturbances accelerate disease progression and are considered critical targets for intervention and treatment (3).

Current nutritional management strategies for CLD are largely standardized and population-based, following a “one-size-fits-all” methodology. In contrast, these strategies fail to adequately address the complex inter-individual variations in genetic background, metabolic status, gut microbiota, and disease staging. These limitations restricts the efficacy of interventions and complicates personalized health management for CLD patients throughout their disease progression (4). In response, the emerging concept of precision nutrition presents a novel approach to the nutritional prevention and management of CLD. Precision nutrition incorporates multi-omics data—such as genomics, proteomics, and metabolomics—alongside patients’ lifestyle factors and nutritional status, this facilitates the development of scientifically rigorous and dynamic dietary guidance and health management plans that are tailored to individual needs (5, 6).

**Figure 1:**
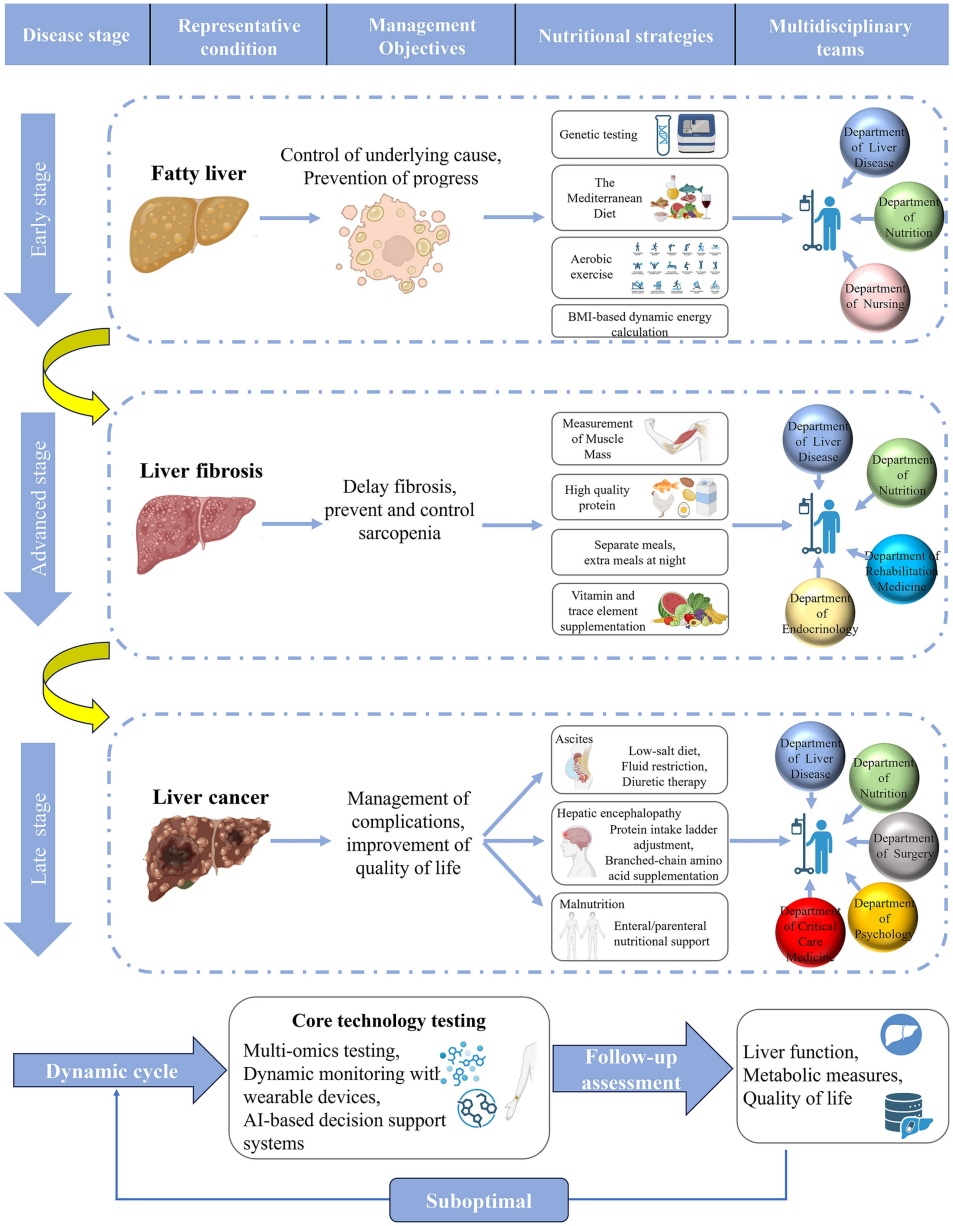
Framework illustrating a whole-cycle precision nutrition intervention for CLD. Created with BioRender.com.

With the continuous advancement of both fundamental and clinical research on precision nutritional interventions in CLD, relevant guidelines and expert consensus statements have been progressively introduced, highlighting the significant potential of this approach to enhance patients’ nutritional and metabolic status, delay disease progression, and improve QoL (7). However, several changes impede the effective integration of precision nutrition into comprehensive health management throughout the entire course of CLD. These challenges include insufficiently mature technology, limited clinical translation pathways, and the urgent need to establish standardized systems. This study aims to systematically review the current state of research, application value, and developmental trends in precision nutrition for the comprehensive management of CLD, thereby providing theoretical and practical references for future research, the development of precision prevention strategies, and the optimization of integrated management systems. As illustrated in Figure 1, the whole-cycle precision nutrition intervention framework offers a comprehensive approach to managing CLD by addressing the unique needs of each patient at various stages of the disease.

## 2 Full-cycle health management of CLD

### 2.1 Definition and theoretical basis

With the advancement of precision nutrition, its essential role in the whole-course health management for CLD patients has gained increasing recognition. Precision nutrition dynamically optimizes nutritional interventions tailored to patients’ genetic backgrounds, metabolic profiles, and lifestyle factors. This approach provides substantial technical and theoretical support for implementing whole-course management models. Consequently, effective whole-course health management of CLD requires the strong support and thorough integration of precision nutrition, which optimizes patient outcomes. Full-cycle health management refers to the continuous implementation of dynamic and individualized comprehensive management measures throughout the complete course of disease progression, encompassing stages such as disease prevention, early diagnosis, standardized treatment, rehabilitation, and long-term follow-up (8, 9). This concept emphasizes a systematic approach to disease management, making it particularly relevant for CLD, which is characterized by a prolonged and distinct disease trajectory, aiming to integrate prevention, treatment, and health promotion (10).

### 2.2 Combined with the concepts of “lifecycle-based management” and “multidisciplinary team”

Full-cycle health management aligns with the principles of lifecycle-based management, which systematically addresses the comprehensive health needs of patients across the entire continuum from wellness and risk exposure to disease onset, progression, and end-stage disease (11). By stratifying risks and implementing timely, stage-specific interventions, this approach has the potential to delay disease progression and improve overall prognosis. In the case of chronic hepatitis B (CHB), management involves not only antiviral therapy but also health education, nutritional support, behavioral counseling, and regular monitoring, with a focus on prevention, long-term follow-up, and end-of-life care when appropriate (12).

The management of CLD requires the support of a multidisciplinary team (MDT). Patients with CLD often face concurrent nutritional, metabolic, psychological, and functional challenges that are not adequately addressed by single-specialty care. An MDT, involving hepatology, nutrition, endocrinology, psychology, rehabilitation, and other relevant specialties, facilitates the development of individualized and coordinated management plans (13). Evidence from patients with MAFLD indicates that multidisciplinary dietary interventions can improve liver enzyme levels at 12 post-intervention and follow-up. Patients in the MDT group experienced an average weight loss of 4.2 kilograms, markedly greater than the 0.6 kilograms observed in the conventional treatment group (*p* < 0.01). Moreover, the intervention group exhibited a lower rate of weight regain (14). Therefore, integrating lifecycle-based management with MDT care provides a structured framework for continuous, individualized, and precise management, enhancing QoL and clinical outcomes in patients with CLD.

### 2.3 Key points of managing CLD at different stages

CLD is typically classified into three stages: early, progressive, and advanced stages. At each stage, management involves standardized protocols, stratified interventions, and a multidisciplinary approach. These steps provide the foundation for comprehensive health management throughout the disease progression.

#### 2.3.1 Early stage

The primary focus of early-stage CLD management, including conditions such as early chronic hepatitis B and nonalcoholic fatty liver disease, is to identify the underlying etiologies, manage risk factors, and enhance patients’ self-management capabilities through health education. Specific interventions involve modifying dietary habits by reducing the consumption of high-calorie, high-fat, and high-sugar foods, increasing the intake of fresh fruits, vegetables, and whole grains, and promoting regular physical activity. Studies indicate that adherence to a low-energy Mediterranean diet, combined with aerobic exercise over 12-months, can substantially decrease hepatic fat content, improve insulin resistance, and delay disease progression (15). In addition, for patients with viral hepatitis, standardized antiviral therapy can effectively suppress viral replication, mitigate hepatic inflammatory responses, and prevent secondary metabolic disorders (16). At this stage, daily energy requirements are calculated using the patient’s body mass index (BMI) and basal metabolic rate. These requirements are then adjusted to support the achievement of a healthy body weight and metabolic balance, which can prevent disease progression (17).

#### 2.3.2 Advanced stage

Upon advancing to the stages of liver fibrosis and compensated cirrhosis, management priorities shift towards slowing disease progression, preventing complications, and maintaining nutritional status (18). During this stage, declining hepatic synthetic function substantially increases the risk of protein–energy malnutrition. Regular assessment of liver function, biochemical markers, and muscle mass is essential for the early detection of sarcopenia (19). Previous studies have demonstrated that supplementation with appropriate amounts of high-quality proteins, such as whey and soy, supports the preservation of muscle mass in cirrhotic patients (20). In addition, supplementation with vitamins, particularly vitamin D and the B-complex vitamins, as well as trace elements such as zinc and selenium, is necessary to prevent deficiencies. Patients with cirrhosis are susceptible to deficiencies of fat-soluble vitamins A and E due to impaired bile secretion, necessitating regular assessment and supplementation of these vitamins (21). Therefore, it is recommended that patients at this stage consume small, frequent meals, avoid prolonged fasting, and incorporate late-evening snacks to reduce nocturnal protein catabolism and improve protein balance (22).

#### 2.3.3 Late stage

In patients with decompensated cirrhosis and hepatocellular carcinoma, management focuses on preventing and treating complications, optimizing the QoL, and providing psychological support (23). Common complications at this stage include ascites, hepatic encephalopathy, and gastrointestinal bleeding. Nutritional strategies should be dynamically adjusted, including enteral or parenteral nutrition as indicated. MDT is essential to protect patient’s physical and psychological well-being (3). Patients with ascites should adhere to a low-sodium diet, whereas those with hepatic encephalopathy require protein intake adjustments according to the stage of the disease. During the initial episode, short-term protein restriction may be considered, and once symptoms improve, protein intake should be gradually restored to 1.2–1.5 g/kg body weight per day (24). If oral intake is inadequate, early initiation of enteral or parenteral nutrition is recommended. For patients with hepatocellular carcinoma, a comprehensive preoperative nutritional risk assessment is essential. Early postoperative initiation of enteral nutrition can promote recovery and reduce the length of hospital stay (25). In addition, a multidisciplinary team—including nutritionists, hepatologists, and psychiatrists—should collaborate to provide precision nutritional and psychological interventions, supporting patient coping and enhancing QoL (22).

Recent recommendations from the Chinese Society of Hepatology and international consensus guidelines suggest that patients with CLD should receive stratified and precision nutritional management at various stages of the disease to meet evolving physiological and metabolic demands throughout disease progression. Comprehensive, stage-specific, and multidisciplinary health management of CLD—including precise nutritional support—aims to prevent disease onset, slow progression, improve outcomes, and enhance patient health. However, traditional stage-based and standardized management strategies benefit most patients; they often overlook complex inter-individual variations. The integration of precision nutrition overcomes this limitation by enabling individualized and intelligent optimization of nutritional interventions, based on multidimensional characteristics such as genetic background, metabolic status, and disease dynamics. This approach enhances the scientific rigor and effectiveness of whole-course management in patients with CLD.

Table 1 presents a comparison of precision nutrition interventions designed for different types and stages of liver disease, emphasizing core objectives, dietary patterns, guideline recommendations, and the supporting evidence for each condition. Due to the descriptive nature of this review, the certainty of the evidence in the table is based on a narrative assessment that incorporates current guidelines, the availability and consistency of clinical evidence, study design, sample size, and the direct relevance of the evidence to CLD populations. Therefore, the “Evidence basis and certainty” column should be interpreted as a guideline-based summary of the supporting evidence, rather than a formal GRADE assessment.

**Table 1:** Comparative analysis of precision nutrition interventions based on liver disease type.

Types of liver disease	Core intervention objectives	Recommended dietary patterns	Recommendations from guidelines	Evidence basis and certainty	References
Metabolic-associated fatty liver disease (MAFLD/NAFLD)	Achieve 5–10% weight loss; improve insulin resistance; reduce hepatic fat deposition; optimize metabolic indicators	Mediterranean diet; limit high-fructose/high-fat foods; polyunsaturated fatty acid fortification; high dietary fiber intake; aerobic exercise for 30 min per day	AASLD guidelines Negative energy balance promotes weight loss; protein: 0.8–1.2 g/kg/ day	High	(26)
Viral hepatitis (B/C)	Support anti-viral treatment; maintain nutritional balance; improve immune function; promote hepatocyte repair	A balanced diet; appropriate amount of high-quality protein; limiting refined carbohydrates; Vitamin B/C/E supplementation; antioxidant fortification	The EASL guidelines recommend: nutritional support based on antiviral therapy to prevent metabolic disorders	High	(12)
Cirrhosis (compensated)	Protein supplementation (1.2–1.5 g/kg/ day); Prevent sarcopenia; Maintain positive nitrogen balance; Micronutrient supplementation	Multiple meals (4–6 meals/day); bedtime snack; whey/soy protein; vitamin D/ zinc/selenium supplementation; Avoid prolonged fasting	ESPEN guidelines recommend: Energy: 28–35 kcal/kg/ day to improve muscle mass and prognosis	High	(22)
Cirrhosis (decompensated stage)	Manage complications comprehensively maintain nutritional status; improve quality of life; provide psychological support	Patients with ascites: low-salt diet; hepatic encephalopathy: graded protein adjustment; enteral/parenteral nutrition support; branched-chain amino acid supplementation; small, frequent meals	ESPEN/AASLD guidelines: multidisciplinary team collaborative management; individualized, dynamic nutritional adjustment	Moderate	(27)

The “Evidence basis and certainty” was categorized as high, moderate, or low through a narrative approach, considering factors such as guideline authority, consistency of available evidence, study design, sample size, and direct relevance to CLD populations. This assessment did not follow a formal GRADE methodology. Furthermore, translated or re-published guideline versions were not graded as independent sources of evidence.

## 3 Theoretical basis and core technology of precision nutrition

### 3.1 Definition and development of precision nutrition

Precision nutrition currently lacks a universally accepted definition. The National Institutes of Health (NIH) defines it as a nutritional management approach that utilizes multidimensional individual-level data to guide dietary assessment and intervention (28). In this review, precision nutrition is defined as a data-driven strategy that integrates multidimensional individual factors, such as genetic background, metabolic phenotype, physiological status, lifestyle, dietary patterns, disease stage, and gut microbiota composition, to inform nutritional assessment and intervention. The objective is to develop safe, context-specific dietary approaches that promote health maintenance and prevent or delay disease progression (5, 6, 29).

Standard nutritional management relies on population- or disease-specific guidelines, tailored with conventional clinical variables such as age, sex, body weight, nutritional status, physical activity, and disease severity. This approach remains a key element in the nutritional care of CLD, particularly for patients with decompensated cirrhosis, where sarcopenia and frailty are often observed. Personalized nutrition further refines dietary recommendations based on the individual’s clinical condition, lifestyle, preferences, and nutritional requirements. Precision nutrition further advances this model by incorporating comprehensive biological data, such as genomics, metabolomics, inflammatory profiles, and gut microbiome characteristics, often utilizing big data analytics and computational tools (30, 31). These biological factors can significantly affect nutrient absorption, utilization, metabolism, and the body’s response to nutritional interventions.

The concept of “precision” was originally derived from precision medicine, as introduced in the 2011 U.S. National Research Council report *Toward Precision Medicine*, and has since been adapted for use in nutrition (5). Unlike conventional nutritional interventions, precision nutrition focuses on biological heterogeneity among individuals, aiming to improve risk assessment and intervention. This approach is particularly relevant in CLD, where patients with similar dietary patterns or clinical diagnoses may exhibit significant differences in steatosis, inflammation, fibrosis progression, and treatment responses.

Recent studies have highlighted that genetic variants, such as those in *PNPLA3*, along with metabolic and microbiome-related markers, may facilitate the identification of high-risk individuals and support the development of targeted dietary interventions (32). However, the available evidence on CLD remains limited, with many findings being associative or derived from broader metabolic research. Therefore, precision nutrition should be considered an emerging and complementary strategy, rather than a replacement for well-established guideline-based nutritional care.

#### 3.1.1 Omics and microecology

The concept of precision nutrition is rooted in advanced fields, including nutrigenomics, metabolomics, and research on gut microbiota. There is significant inter-individual variability in glycemic and lipid responses to identical food, due to interactions among genetic factors, gut microbiota composition, and lifestyle. A comprehensive cohort study conducted by Zeevi et al. in Israel analyzed clinical data, dietary behaviors, genotypes, gut microbiota, and blood biomarkers from over 800 participants revealing considerable differences in postprandial glycemic responses among individuals. Utilizing multi-omics data, the research team developed a personalized dietary intervention model that accurately predicts glycemic responses and provides tailored dietary recommendations. After 1 week of precision dietary intervention, the participants showed significantly reduced postprandial glycemic fluctuations, improved glycemic control, and better metabolic syndrome-related parameters (33). Similar multi-omics integration strategies are increasingly being applied in the field of CLD. Studies have demonstrated that gut microbiota dysbiosis in patients with liver disease is often associated with inflammation and the progression of hepatic fibrosis. Assessing gut microbiota characteristics and implementing targeted modulation can intervene in metabolic disturbances related to CLD, improving liver function and inflammatory responses (34). These advanced technologies provide a robust, data-driven, and theoretical foundation for the practical application of precision nutrition in the comprehensive health management of patients with CLD throughout the course of their disease.

### 3.2 Key technologies

#### 3.2.1 Molecular biology and omics technology

Molecular biology and omics technologies provide the foundation for understanding the physiological and nutritional differences in how individuals respond to various stimuli. High-throughput platforms, such as genomics, transcriptomics, proteomics, metabolomics, and microbiomics, enable researchers to systematically investigate how individuals respond to specific nutrients or dietary patterns (35). Metabolomics analyses detect subtle alterations in energy, lipid, and amino acid metabolic pathways in patients with CLD. This supports the identification of early metabolic disturbances and potential nutritional intervention targets (36). Recent studies have analyzed the composition of gut microbiota and metabolic profiles of patients with cirrhosis, revealing correlations between specific microbial shifts and the risk of hepatic encephalopathy. These results support the development of individualized interventions, including probiotics and prebiotics (37). In patients with NAFLD carrying high-risk PNPLA3 genotypes, combining gene testing with nutritional risk assessment allows for the precise formulation of dietary recommendations—such as low-fat, polyunsaturated fatty acid-rich, and high-fiber diets—to improve disease management (38).

#### 3.2.2 Individual dietary assessment and dynamic monitoring tools

High-resolution individualized dietary assessment and dynamic tracking are essential for optimizing precision nutritional interventions. Recent advances in nutritional informatics, continuous metabolic monitoring, and mobile internet technologies have made it possible to manage nutrition in both clinical settings and at home (39). Continuous glucose monitoring (CGM) systems track blood glucose fluctuations in real-time under various dietary patterns, enabling physicians and patients to make timely adjustments to their dietary plans. This capability is particularly significant for individuals with CLD and glucose metabolism disorders (40). Intelligent dietary applications and wearable devices automatically record daily dietary intake, physical activity, and body weight. This supports comprehensive visualization and management of nutrient intake and energy expenditure. Healthcare teams can use app-recorded dietary data and physiological monitoring to dynamically adjust protein and energy intake for patients with CLD, effectively preventing malnutrition and sarcopenia (41).

#### 3.2.3 Application of big data and AI in nutrition management

The integration of big data and AI has significantly advanced precision nutrition, enhancing data integration and decision-support capabilities. Machine learning and deep learning algorithms can identify key determinants of nutritional responses and predict disease risk using data sources, including clinical records, multi-omics profiles, and lifestyle factors information. AI models can predict blood glucose and lipid responses in patients with CLD to specific dietary interventions, helping physicians develop precision nutritional plans (33). Furthermore, AI systems continuously learn from new patient data, provide real-time recommendations for optimizing nutritional interventions, and support adaptive feedback mechanisms to improve management efficiency (42). Combining molecular omics testing, individualized dynamic monitoring, and big data/AI platforms substantially increases the effectiveness of precision nutrition in managing complex diseases, such as CLD. This approach provides personalized, dynamic, and evidence-based nutritional management for chronic diseases, thereby enhancing patient outcomes and improving QoL.

## 4 Research progress and application status of precision nutritional interventions for CLD

### 4.1 Application of precision nutrition in different CLD

Precision nutrition is essential for the prevention, treatment, and management of CLD. Nutritional strategies should be differentiated and individualized based on the specific type and stage of CLD (22). Precision nutrition relies on dynamic nutritional risk assessment and individualized dietary management. Tools such as the Nutritional Risk Screening (NRS)-2002 and the Global Leadership Initiative on Malnutrition (GLIM) are used to regularly evaluate nutritional status and formulate interventions based on disease characteristics. Recently, the integration of multi-omics analyses—including genomics, metabolomics, and gut microbiome profiling, with dynamic nutritional monitoring and AI-driven data integration has advanced precision nutrition interventions. These developments allow for more individualized and dynamic nutritional management, which can optimize health outcomes in patients with CLD (33, 43).

Nutritional interventions for CLD caused by viruses, such as hepatitis B and C, involve adjusting energy, protein, and micronutrient intake based on antiviral therapy guidelines (16). These interventions aim to enhance immune function and promote hepatic recovery. They also regulate fat and simple carbohydrate intake to minimize metabolic disturbances and reduce the risk of malnutrition. Genomic and metabolomic testing identify patient-specific nutritional needs among patients with viral liver diseases, enabling targeted supplementation of antioxidants and essential nutrients. This individualized approach supports real-time monitoring of nutritional risks and the optimization of intervention strategies (44, 45).

Effective management of MAFLD requires optimizing dietary composition, maintaining energy balance, and achieving effective weight control. According to the European Association for the Study of the Liver (EASL) international guidelines, energy intake should be adjusted to achieve a 5–10% reduction in body weight. Protein intake should be maintained at 0.8–1.2 g/kg body weight per day. Consumption of food high in sugar and fat, particularly trans fats and fructose, should be limited (8). Adherence to healthy dietary patterns, such as the Mediterranean diet or the Dietary Approaches to Stop Hypertension (DASH) diet, in combination with behavioral and exercise interventions, has been shown to improve hepatic fat content and metabolic parameters (15, 46).

Patients with cirrhosis, especially those in the decompensated stage, often experience malnutrition and sarcopenia, requiring dynamic adjustments of energy intake (28–35 kcal/kg of body weight/day) and high-quality protein intake (1.2–1.5 g/kg of body weight/day). Small, frequent meals and late-evening snacks are encouraged to promote protein anabolism (47). Regular assessment and early intervention for sarcopenia can significantly improve patient outcomes.

### 4.2 Evidence-based clinical study of precision nutrition intervention

Clinical evidence supporting precision nutrition in CLD is increasing; however, it remains heterogeneous and largely preliminary. Zeevi et al. (33) developed a multi-omics-based model to predict individualized postprandial glycemic responses (PPGR), providing proof of concept that data-driven dietary strategies may enhance metabolic risk stratification. In CLD populations, Hu et al. demonstrated that dynamic nutritional assessments, combined with individualized dietary interventions, improved nutritional status and QoL among Chinese patients with CLD (4). Additionally, a study from Mexico indicated potential benefits of personalized nutrition on selected liver-related and metabolic indicators; however, due to limitations in study design and sample size, these findings should be viewed as exploratory rather than conclusive (48).

It is essential to distinguish direct CLD evidence from studies conducted in other populations. For example, the BREAst Cancer Personalised NuTrition (BREACPNT) study is a randomized clinical trial (RCT) protocol in breast cancer (BC) survivors undergoing endocrine therapy, designed to evaluate an algorithm-based personalized meal-planning approach to improve PPGR (49). Since this study presents a protocol rather than clinical outcomes and is not focused on CLD-specific outcomes, it should be cited as an example of algorithm-supported personalized nutrition methodology, not as evidence for efficacy in CLD. Overall, current findings support the feasibility and potential clinical relevance of precision nutrition; however, robust, prospective, and CLD-specific interventional studies are necessary to establish its efficacy, safety, and long-term value.

### 4.3 Practice model of precision nutrition management

**Figure 2:**
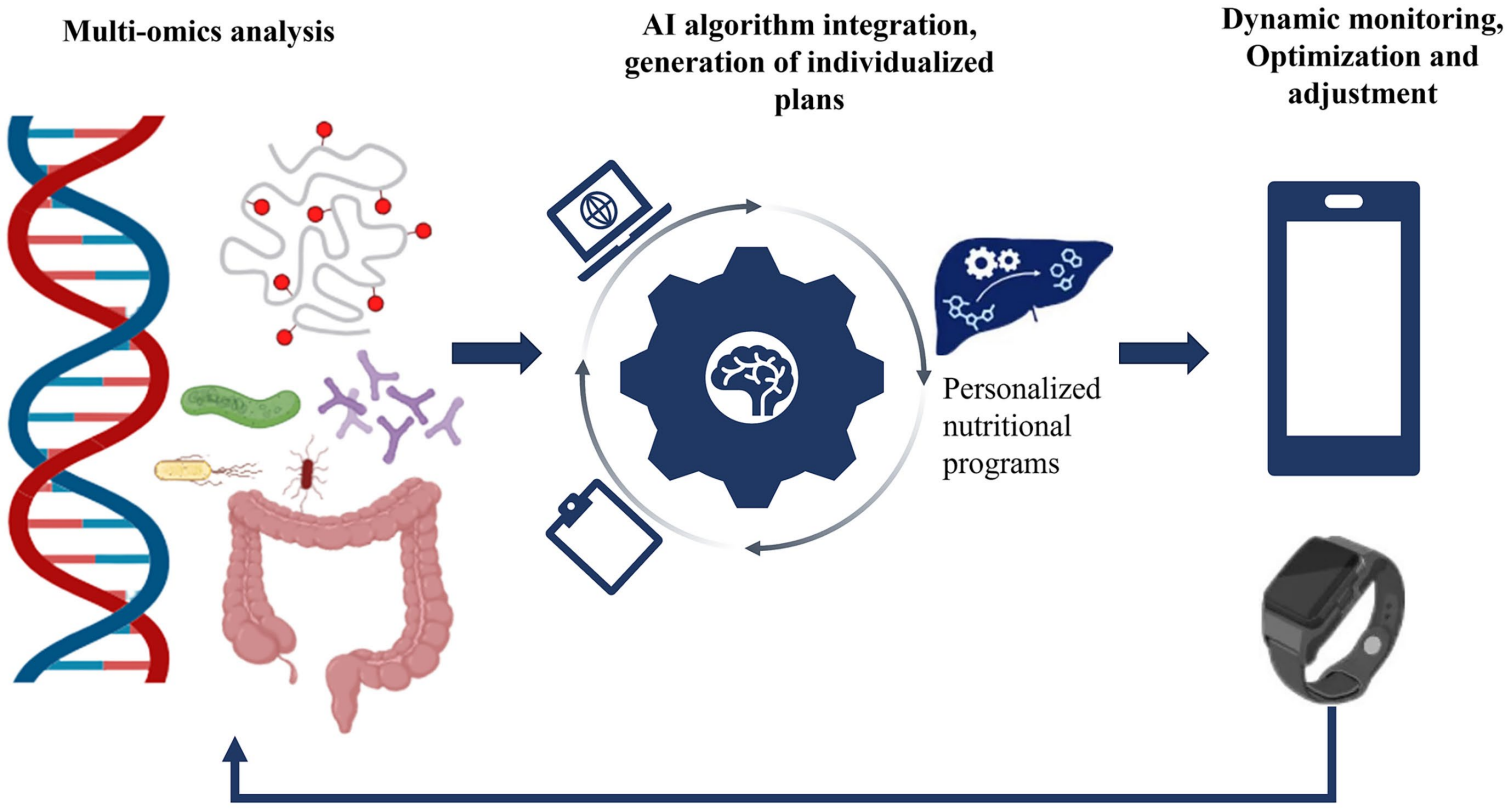
Integrated framework for the core technologies of precision nutrition. Some graphical elements were created with BioRender.com.

Precision nutrition management emphasizes the importance of MDT, This approach involves experts from hepatology, nutrition, endocrinology, psychology, and exercise rehabilitation to formulate and dynamically adjust precision nutritional plans (50). Integrating digital health management tools, such as wearable devices, smart applications, and remote monitoring platforms, has been crucial for monitoring dietary behaviors, nutritional risk alerts, and follow-up management for patients with CLD. This approach has significantly enhanced the scientific foundation and adherence to management protocols. In clinical practice, implementing strict nutritional management pathways and sharing case studies through MDT consultations, patient education, and individual case follow-up have extensively promoted the implementation and dissemination of the precision nutrition concept. As shown in Figure 2, the integrated framework for core technologies of precision nutrition outlines the essential components and methodologies critical for its application in chronic liver disease management.

## 5 Existing problems and challenges

Precision nutrition has emerged as a promising area of interest in the management of CLD. However, current evidence is insufficient to support its widespread clinical implementation. Therefore, it is crucial to distinguish between established evidence and emerging findings in this field. To date, CLD-specific intervention studies remain limited, and many proposed strategies are based on observational studies, mechanistic research, or data from broader metabolic disease populations, which limits causal inference and disease-specific generalizability. Existing studies on CLD demonstrate considerable variation in factors such as underlying causes, disease stages, baseline nutritional status, intervention components, outcome definitions, and follow-up duration. Furthermore, small sample sizes, short observation periods, limited randomization, inadequate control of confounders, and potential biases in selection or reporting reduce confidence in conclusions regarding long-term efficacy, safety, adherence, and cost-effectiveness (15, 22). Therefore, various precision nutrition methodologies are currently considered promising; however, they have not yet been definitively proven.

The implementation of precision nutrition in clinical practice faces multiple barriers. It often relies on omics technologies, bioinformatics analysis, and multidisciplinary interpretation; however, the costs associated with testing, equipment, data storage, and analytical expertise remain high, especially in primary care and resource-limited settings (33). Moreover, many biomarkers lack clinical validation, standardized thresholds, and established decision-making frameworks. Healthcare professionals require additional training to interpret precision nutrition data and to integrate individualized recommendations into the management of CLD. At the patient level, adherence to personalized dietary plans depends on economic, cultural, behavioral, and disease-related factors (13).

In conclusion, precision nutrition requires a balance between individualized decision-making and standardized, scalable clinical workflows. Currently, there is a lack of standardized protocols for nutritional assessment, intervention adjustments, outcome evaluation, and follow-up. Future research should focus on adequately powered, prospective CLD-specific trials that include standardized endpoints, longer follow-up periods, transparent reporting, bias assessments, and real-world evaluations of feasibility, safety, adherence, and cost-effectiveness.

## 6 Summary and prospect

Precision nutrition holds promise as an evolving framework for the comprehensive management of CLD. By integrating nutritional assessments with clinical characteristics, disease stage, metabolic status, lifestyle factors, and, when feasible, omics and digital health data, this approach has the potential to enable more individualized dietary interventions and patient-centered care. However, the current clinical value of precision nutrition must be interpreted with caution. The available evidence is limited by a small number of CLD-specific intervention studies, heterogeneity in study populations and intervention designs, small sample sizes, short follow-up periods, and inconsistent outcome measures. Therefore, most precision nutrition strategies should be considered as emerging approaches rather than established clinical standards.

Future research should prioritize well-designed, prospective, multicenter, and real-world studies to evaluate the efficacy, safety, adherence, cost-effectiveness, and long-term clinical outcomes of nutritional interventions for CLD across different etiologies and disease stages. The development of standardized frameworks for nutritional assessment, intervention adjustment, endpoint selection, and follow-up is essential to improve reproducibility and facilitate clinical translation. The effective implementation of feasible care pathways will require multidisciplinary collaboration among hepatologists, dietitians, data scientists, and primary care providers. Emerging technologies, such as artificial intelligence (AI), remote monitoring, and clinical decision support systems, hold the potential to enhance scalability. However, these approaches require rigorous validation, data standardization, robust privacy protection, and careful consideration of health equity. Overall, the broader application of precision nutrition in CLD will rely on stronger evidence and practical implementation models.
